# Notes on the Mayo Clinic

**Published:** 1910-12

**Authors:** R. M. Harbin

**Affiliations:** Rome, Ga.


### NOTES ON THE MAYO CLINIC.

By R. M. Harbin, M. D., Rome, Ga.

Perhaps no phenomenon in the history of surgery excites more curious interest than that which centers around the Mayo clinic. A Pullman car is in waiting each night in Chicago for the pilgrimage of visiting surgeons and the many patients en route to the world's Mecca of surgery, Rochester, Minn., a small city of 8,000 inhabitants. According to the register about 3,000 surgeons from all countries visit this clinic each year and are greeted with the utmost hospitality, and the effort to show you is always in evidence. The clinic is directed by the pioneers of the modern technique of surgery of the upper abdomen and the thyroid gland, and as evidence of the rapid growth of the major surgery done at St. Mary's Hospital in 1893, there were admitted patients 405 and 41 abdominal sections. while in 1909 there were admitted 4.809 and 3.762 abdominal sections. It is readily seen that the northwest has the largest clinic in the world for major surgery. The noted surgeon, Dr. W. W. Keen, of Philadelphia, was operated there for diverticulitis of the sigmoid, in November, and requested that his case be demonstrated to the visiting surgeons. The benefits of this clinic are greatly enhanced by the Surgeons' Club, which every visiting surgeon is expected to join. Reporters are appointed for each operating room for each day during the week. The clinic begins at 7:30 A. M., lasts until 1 P. M., during which time twenty to thirty operations are performed, most of which belonging to the major class. The Surgeons' Club meets at 3 P. M., and the reporters read their notes, which are freely discussed. While only a nominal fee is paid to the club, personally, I am of the opinion that almost every member would esteem it a privilege to pay a much larger fee to facilitate the equipment for evening demonstrations and lectures on the part of the staff as well as distinguished visitors. The equipment for scientific work at St. Mary's hospital has greatly enlarged its facilities and combined with the great amount of practical work that is being done here, gives the institution a commanding position in medical science.

As evidence of the progressive spirit of the institution, one or more members of the staff are constantly visiting the best clinics at home and abroad and notes of these observations are read at the staff meetings, as well as the condensed reviews of medical literature. Dr. W. J. Mayo kindly invited me to attend one of these meetings and among others, he made his report of a recent trip to the Eastern Clinics. He mentioned having studied the experiments of Carrell of the Rockefeller Institute, in proving that peritoneum and kidney tissue could be made to grow artificially in the serum of the animal in question. He also referred to the operation of Dr. Willy Meyer, of N. Y., in opening the cardia for the relief of cardio-spasm.

A peculiar fact relates to this region of the body, that ordinary methods of aseptic technique are not sufficient to prevent infection because of the great vulnerability of the tissues. He said Dr. Meyer had ligated the pneumogastric nerves twice, with one recovery. The claim of Le Conte, of Philadelphia, that the entire removal of the shaft of the tibia for osteo-myelitis could be followed by replacement was discussed. Dr. Mayo exultingly remarked that Dr. J. B. Deaver had become converted to the Oschner treatment of appendicitis.

At the Mayo clinic ether is the anaesthetic of choice, as chloroform was induced only once last year. It is claimed that no death has resulted from anesthetic per se in over 37,000 administrations. Nor has there occurred a case of ether pneumonia. The drop method on an open mask is the only apparatus used and the anesthetists are nurses. Morphine precedes the ether only in stomach operations and in thyroidectomies it is combined with atrophine.

The patient is given 2½ oz. castor oil at 4 P. M., and after a light supper an enema is administered at 4 A. M. It is advised that patients walk to the operating room as it is reassuring. The abdomen having been shaved is scrubbed on the operating table and then bathed with Harrington's solution (Mercuric chloride .8 Hydrochloric Acid .60, alcohol 700, water 240), and then painted with iodine. Too much scrubbing by macerating the skin favors infection.

In the last six months there was only one infection in 600 wounds. In the event of infection the sutures are merely loosened and a compress of a saturated solution of boric acid and alcohol is applied and over this a hot water bag. Probing is believed to be dangerous. Knives are kept sterilized in a 10% solution of lysol and gloves are boiled and emersed in a 1 to 5,000 bichloride solution.

In every case the incision is large enough to admit the hand so that no lesion can escape discovery.

A 10%callargol solution is used for X-ray photography and in the corner of the operathing room there is an illuminated chamber for displaying the plates.

The importance of the specific gravity of urine as an index to safety in operations is emphasized in the aged. When it persists as low as 1,006 or 1,008, operation is positively contra-indicated. But by forcing liquids and removing the residual urine in the bladder, the specific gravity can be raised. A moderate amount of albumen and castes are not regarded seriously. It is claimed that the X-ray will show stones in 97% in the kidney parenchyma, and 90% in the lower ureter in the cases where stones arê present.

The importance of Beck's bismuth paste (bismuth subnitrate 30, vaseline 60, soft paraffine 5, white wax 5), as diagnostic and therapeutic ,is emphasized, and bismuth paste will cure nearly all small sinus cavities.

In the replacement of intestines the omentum is always carefully folded over them.

SUTURES.

Silk is never used at the Mayo clinic and in the adjustment of sutures tension is carefully avoided as a cause of necrosis and infection. The layer suture with the Bartlett catgut is the routine method, and in hemiotomies chromic catgut is used. About three silk worm gut sutures are applied loosely in the form of the figure 8 to take of tension. The suture is passed down through the skin of one side and then down through the muscular layers of the opposite side and up again through the muscular layers and across again up through the skin on the other side, and then tied loosely and removed in two weeks. The edges of the skin are approximated by a loop suture of horse hair.

Linen is the material used for intestinal suture.

In every drainage case plain catgut is used, for the chromic gut will provoke a sinus. Where fat is excessive there is danger of hematoma and a small drainage should be used. Cotton as a dressing is not used except in drainage cases. Over the gauze are applied adhesive strips, and over this a large roller of gauze is wound around the body several times.

APPENDICITIS.

The Mayos do not believe the appendix has any function. The dyspeptic type of appendicitis gives a history of long periods of attacks. While no pain is referred to McBurney's point, there is usually tenderness, and pain in the lower abdomen always means appendicitis. Nausea is more characteristic than vomiting. The presence of fecal concretions indicate appendectomy.

Half of the appendices become obliterated after the age of 50 years. In the acute attack pain precedes fever.

Infections behind the ascending colon are peculiarly troublesome and require stab drainage in the right flank.

The appendix is not actively sought in abscess cases and usually undergoes degeneration.

Unnecessary drainage is not considered at all dangerous. Fat mesenteries of the appendix are crushed before tying, and the stump is always buried with a purse-string of linen. No cautery is used.

The purse-string is introduced before the stump is cut. There have been three hemorrhages from slipping ligatures at this clinic.

### Lane's Kink.

It is to be noted that a considerable number of cases of appndectomy continue to suffer with pain which has been supposed to be due to adhesions. The importance of searching for Lane's kink is stressed as explaining this phenomenon. The last three or four inches of ileum from a downward pulling is made to roll on its mesentery, forming an adhesion and thus produces a kink in the gut. This should be carefully inspected and if found the adhesions should be separated.

### Gall Bladder.

In 1909 there were 589 ball gladder operations at the Mayo Clinic. The gall bladder is compressed with one hand in order to determine the patency of the cystic duct.

In every one of the 74 cases of cancer in the gall bladder stones were found.

Four-fifths of the gall stones occur in women, 80% of which occurring after childbirth or typhoid fever. The colon bacillus is the usual form of infection of the gall bladder. 5% of stones givs no symptoms.

When bile is vomited it usually comes from the gall bladder. Sudden pain in the epigastric region with no loss of flesh points to the gall bladder. The Mayos never saw gall stones reform but once.

Adhesions between the gall bladder, stomach and duodenum, should be separated, while those with the omentum and transverse colon should remain undisturbed. A red gall bladder usually indicates empyema and after suturing the tube by the purse-string method another spit tube with gauge is used to drain outside the gall bladder.

It is not considered necessary to anchor the gall bladder to the parietal peritoneum.

When the tube is in place is is made to drain in a bottle which is enveloped in the dressing.

5% of the cases of infected gall bladder require cystectomy.

### Ulcer and Cancer.

73% of cancers give a definite history of ulcer lasting 3 to 15 years, and the average duration of symptoms of cancer of the stomach is seven months. 95% of tumors of the stomach are cancerous, and tumors that are high and to be left are inoperable.

Remnant food in the stomach indicates a surgical condition. The pathologist has demonstrated that 51% of cancers were developed on the edges of an ulcer. The presence of blood in feces has litle or no significance, and frequently occurs in the absence of ulcer and cancer. Vomiting relieves pain in cancer, but fails to do so in ulcer and eating relieves pain in ulcer. Operations on the stomach are not advised until the stomach shows a good acidity.

The appearances of the peritoneal covering of ulcers are very distinct. In inverting a duodenal ulcer a patch of omentum is sewed on and then a posterior gastro enterostomy is performed.

### Goitre.

It is estimated by the assistant to Dr. C. H. Mayo that this year will give a record of 600 thyroidectomies, while the record of Kocher, of Berne, Switzreland, will give 400.

The size of the tumor is no index to the amount of toxemic disturbances. In all cases of exophthalmic goitre there is an increase of the parenchyma of thyroid gland, thus causing an increase of the internal secretion. and if it does not kill it reverts back to simple goitre. This increased internal secretion first causes a rapid heart and later brings about degenerative changes in other organs. which are shown by irregular heart. pulsating jugulars, valvular insufficiency, etc., which cause death. Thyroidectomy interrupts this generative process. The enlarged thyroid receives more blood than the brain and hemostasis is the watchword of operative procedure.

The parathyroids are supplied by the inferior vessels and are not disturbed.

A serum treatment has been used with partial success. An infusion of an exsected goitre is injected into the rabbit's blood, and after a series of such inoculations a serum is prepared. Quinine and belladonna have also been used in the treatment of these cases.

Dr. C. H. Mayo emphasizes the importance of delivering these patients to the surgeon before serious degenerative changes have occurred. He discussed the frequent occurrence of goitre in dogs and said a medical friend of his cured a horse of dyspnoea by removing the thyroid. He further said that his pathologist could tell the symptoms of the patient before thyroidectomy from an examination of the gland.

